# Finding a BETTER way: A qualitative study exploring the prevention practitioner intervention to improve chronic disease prevention and screening in family practice

**DOI:** 10.1186/1471-2296-15-66

**Published:** 2014-04-11

**Authors:** Donna Patricia Manca, Michelle Greiver, June C Carroll, Ginetta Salvalaggio, Andrew Cave, Jess Rogers, James Pencharz, Carolina Aguilar, Rebekah Barrett, Shelley Bible, Eva Grunfeld

**Affiliations:** 1 Department of Family Medicine, University of Alberta, University of Alberta, 901 College Plaza, Edmonton, Alberta, T6G 2C8, Canada; 2 Covenant Health, Grey Nuns Community Hospital, 1100 Youville Drive NW, Edmonton, Alberta, T6L 5X8, Canada; 3 Department of Family and Community Medicine, University of Toronto, 500 University Avenue, Toronto, Ontario, M5G 1 V7, Canada; 4 North York Family Health Team, 240 Duncan Mill, North York, Toronto, Ontario, M3B 1Z4, Canada; 5 Department of Family and Community Medicine, North York General Hospital, University of Toronto, 4001 Leslie Street, Toronto, Ontario, M2K 1E1, Canada; 6 Department of Family Medicine Mount Sinai Hospital, University of Toronto, 600 University Ave, Toronto, Ontario, M5G 1X5, Canada; 7 Credit Valley Family Health Team, 2300 Eglinton Avenue West, Mississauga, Ontario, L5M 2V8, Canada; 8 Centre for Effective Practice, 203 College Street, S 24 Suite 402, Toronto, M5T 1P9, Canada; 9 Taddle Creek Family Health Team, 790 Bay St #306, Toronto, Ontario, M5G 1 N8, Canada; 10 Ontario Institute for Cancer Research, Toronto, Ontario, Canada

**Keywords:** Chronic disease, Prevention, Screening, Facilitation, Patient navigator

## Abstract

**Background:**

Our randomized controlled trial (The BETTER Trial) found that training a clinician to become a Prevention Practitioner (PP) in family practices improved chronic disease prevention and screening (CDPS). PPs were trained on CDPS and provided prevention prescriptions tailored to participating patients. For this embedded qualitative study, we explored perceptions of this new role to understand the PP intervention.

**Methods:**

We used grounded theory methodology and purposefully sampled participants involved in any capacity with the BETTER Trial. Two physicians and one coordinator in each of two cities (Toronto, Ontario and Edmonton, Alberta) conducted eight individual semi-structured interviews and seven focus groups. We used an interview guide and documented research activities through an audit trail, journals, field notes and memos. We analyzed the data using the constant comparative method throughout open coding followed by theoretical coding.

**Results:**

A framework and process involving external and internal practice facilitation using the new role of PP was thought to impact CDPS. The PP facilitated CDPS through on-going relationships with patients and practice team members. Key components included: 1) approaching CDPS in a comprehensive manner, 2) an individualized and personalized approach at multiple levels, 3) integrated continuity that included linking the patients and practices to CPDS resources, and 4) adaptability to different practices and settings.

**Conclusions:**

The BETTER framework and key components are described as impacting CDPS through a process that involved a new role, the PP. The introduction of a novel role of a clinician within the primary care practice with skills in CDPS could appropriately address gaps in prevention and screening.

## Background

Chronic conditions affect at least one third of Canadians with their prevalence increasing steadily [1,2]. They have a substantial impact on the use of healthcare services [1]. Family practice is an ideal setting for most chronic disease prevention and screening (CDPS) actions and there are several evidence-based tools and strategies available to improve CDPS, but they are inconsistently applied [3-17]. Examples include the use of electronic medical records (EMR), reminder systems [11], evidence-based guidelines and tools for CDPS actions, patient targeted interventions such as self-management tools [8-10,12,13], and practice-based quality improvement strategies such as practice facilitators [4,6,7,14-16,18].

The Building on Existing Tools to Improve Chronic Disease Prevention and Screening in Family Practice (BETTER) Trial is one example of a successful approach for CDPS that built on existing evidence and applied it in clinical practice [19]. The purpose of the BETTER Trial was to determine whether CDPS for diabetes, cancer (colon, cervical and breast), heart disease and their associated lifestyle risk factors could be improved in the family practice setting using: 1) a practice-level intervention, and/or 2) a Prevention Practitioner (PP) intervention through a prevention visit with a specially trained existing member of the family practice team, the Prevention Practitioner (PP) [19]. The primary outcomes included patient screening activity and health behavior change as measured by the Summary Quality Index (SQUID), a composite index of CDPS [20], and adherence at follow-up (~7 months) after the prevention visit. Full details of the BETTER Trial are published elsewhere [19]. We report here the qualitative strand conducted to understand the contextual factors associated with the positive effect of the PP role within the Better Trial.

The PPs were interdisciplinary health care providers within the family practices and included: registered nurses, a licensed practical nurse, nurse practitioners, and a registered dietitian. Patients aged 40–65 years were invited to participate in the structured PP intervention by completing a health survey and then attending a visit with the PP. Through a rigorous evidence-review process, evidence based clinical guidelines were identified for inclusion in the BETTER CDPS tool kit [21]. The PP was trained in CDPS including: appropriate prevention and screening actions, practice and patient resources to be used during patient encounters, additional CDPS patient resources within and outside the practice, motivational interviewing and shared decision making.

The PP reviewed the patients’ health surveys and medical records to identify preventive actions that patients were eligible to receive. At the time of the visit, through a process of shared decision making and motivational interviewing, a written personalized prescription with prevention and screening goals was tailored to the patient. A significant improvement in the SQUID outcome was observed 7 months later in the PP intervention group [19], demonstrating the effectiveness of this approach.

This qualitative study was conducted with the objective of better understanding the PP intervention in the BETTER Trial described above, including the development of the PP role, perceived barriers, facilitators, benefits and disadvantages, and of exploring the feasibility and sustainability of this approach for CDPS.

## Methods

The BETTER Trial involved four family practice settings in Edmonton, Alberta and four in Toronto, Ontario. It was a mixed methods project that included a pragmatic cluster randomized controlled trial [19] with a qualitative strand. Since our aim for the qualitative strand was to develop an understanding of the PP intervention, this type of question was best suited to a grounded theory (sociology) approach [22-24]. Therefore, an embedded mixed-methods design [25] was used to understand the PP intervention and to inform the future dissemination and implementation of the approach used in the BETTER Trial.

### Participants

To minimize biasing participants, interviews were conducted before the dissemination of the positive results to the participants. For the qualitative strand, we sought perceptions from the PP’s, participating clinicians and administrators. Individuals were eligible to participate from all of the BETTER sites or if they were involved in the BETTER Trial in any capacity. Purposeful sampling was used to recruit participants including PPs, study personnel, clinic support personnel, organizational administrators, intervention and control physicians, other clinic physicians not involved in the project, and clinicians who worked in the clinic. Physicians were selected based on the following characteristics: gender, years in practice, community or academic setting, type of EMR used, physicians with and without patients participating in the BETTER Trial. Potential participants received a detailed letter of information describing the project and inviting them to participate. Both the interviews and focus groups were conducted in person at which time we obtained written informed consent.

The qualitative study, including the recruitment and consent process, received ethics approval from the University of Alberta (Health Research Ethics Board Pro00026547) and the University of Toronto (Health Science Research Ethics Board # 27125) in December 2011. The Trial registration number of the original BETTER RCT was ISRCTN07170460.

### Data collection

Data collection involved individual semi-structured interviews and focus groups (FG) conducted by two BETTER Trial physician investigators and one research coordinator in each city (Edmonton and Toronto). Semi-structured interviews were offered to key informants, physicians or policy personnel when a more candid response was sought. The family physician researchers had expertise in qualitative research (DM, AC, MG, JC) and the research coordinators were non-clinicians (CA, LP). A physician attended each interview since physician participants may be more candid in their response when an interviewer is a fellow physician [26]. A minimum of one physician and one coordinator attended each interview to provide a clinical and a non-clinical perspective. With minimum of two researchers attending the interviews, one was able to focus on leading and maintaining the discussion, while the other focused on recording notes and observations. An audit trail was kept for the entire study and consisted of a step-by-step chronological accounting of the project activities including interviews, discussions and decisions.

Each investigator also captured three types of data:

1) Field notes taken immediately after each interview/FG described general observations such as how people were positioned and non-verbal communication, ideas on possible linkages, potential biases, areas requiring further exploration, reflections on the interview guide, thoughts on sampling and who to approach for further clarification of information.

2) Journals documented a personal audit trail that included evolving perceptions, understandings, potential biases and contrary information that needed further exploration.

3) Memos are fundamental to grounded theory [22,27] and were written throughout the research process. Memos included thoughts on developing ideas, relationships between ideas, and evolving theory.

An interview guide was tested on non-study participants to ensure all areas were captured. The interviews were audio recorded and transcribed. The interviews explored the BETTER PP intervention including perceptions on feasibility and sustainability of the model for CDPS. Two main guiding questions elicited the participants’ stories followed by open ended questions and focused prompts when needed (Additional file 1: Interview guide):

### Analysis

The analysis was conducted using techniques informed by grounded theory methods including the constant comparison method, [22,27,28] which also involved seeking outliers and alternative explanations. The interviews were transcribed verbatim and initially read through and audio-reviewed. Transcripts were then coded line by line with each idea given a name or phrase summarizing the main concept by each investigator independently. The investigators involved in the analysis and coding included six family physicians (DM, AC, GS, MG, JC, JP) and two non-clinicians (CA, LP) (four in Alberta and four in Ontario). This initial coding was reviewed at a face-to-face team meeting to develop some consensus on the codes. The codes were then grouped according to their properties and types into categories and overarching themes. Relationships between different ideas began to emerge and were written as memos. Initially the memos were brief descriptions and early conceptualizations. Through the sorting and resorting of the memos into different groupings and constantly comparing how each memo was related to another [22] the researchers developed a better understanding of the relationships between different concepts and categories, giving the categories dimension and position within the evolving understanding of the situation [28]. Through this evolving understanding the memos become more extensive as they integrated the ideas from earlier memos generating higher-level memos that moved from a descriptive to a conceptual understanding of the evolving framework [22,27].

Each investigator independently drafted journals, and memos of their understandings and themes. These were posted on a secure website and shared with the team. The lead investigator (DM) collected and sorted the information and then posted the collated ideas, themes and categories on the website for review and comments from the team. As the researchers became immersed in coding, a better understanding was developed and this was reflected in higher-level memos [22,27]. At this point a teleconference was held with the research team to develop consensus on evolving themes and hypotheses and future directions to take when re-reviewing the transcripts to capture higher-level memos and conduct selective coding.

The codes were managed manually during the initial open coding and thematic development phase. With a rough understanding of concepts and themes N-Vivo 10 software was then used by DM to further review and capture the evolving understanding and selective coding. The second round of memos were collected and sorted and a framework was developed. A second teleconference was held with the research team to review and refine the evolving framework. The analysis and interpretation was further refined and validated through triangulation with two key informants (PPs).

### Rigor of study methods

Several methods were used to ensure the credibility and consistency or transferability of our findings [23,29-32]. Primarily, grounded theory is best suited to address our objective of developing an understanding of a process [22-24,31]. Researcher reflexivity is also an important component [29,30] and to reduce bias, clinicians and non-clinicians were involved in the interviews and in the analysis. The research team met and questioned each other’s findings and preconceptions and looked to the data for further explanations.

We sought a diverse sample to identify and describe common patterns that transcend a focused sample, providing themes that were common to all [31,33]. Theoretical sampling was used to identify participants from the potential informants since a constant comparative method was used to guide future interviews, that is, we sought data that would challenge and support our evolving understanding, seeking exceptions and looking for variation [27,33].

The research team communicated through face-to-face meetings, emails and through documents posted on a secure website. The use of an interview guide ensured completeness, reduced systematic variance, and assisted with the ability to compare results using the constant comparative method [34]. The interviewers’ field notes collected observations not captured in audio recordings, ensuring comprehensiveness as a form of triangulation, whereby data is gathered from more than one source [24,29,30]. Triangulation was also achieved through theoretical sampling whereby differing perspectives were sought [27,30]. Coding the interviews independently and then meeting regularly to review and compare themes and concepts generated improved rigor and credibility. An audit trail was created throughout the data collection and analysis stages to help with the constant comparison of the data. The information generated met Glaser’s four criteria for judging grounded theory, including fit, relevance, workability and modifiability [27]. Furthermore, respondent validation, or “member checking”, helped to ensure that the interpretations were accurate [24,29,30].

Interviews were conducted until saturation was achieved to ensure that the data categories were dense and the relationship between categories were well established and validated and that no new or relevant data was being obtained; and that all our avenues and leads had been followed and the description was complete [27].

## Results

A total of 45 individuals participated in Alberta and Ontario, including five men and 40 women (refer to Table 1). Individual interviews were held with eight key informants, four in Ontario and four in Alberta. These individuals included administrators, family physicians, and a PP. A total of seven focus groups were conducted at the primary care sites participating in the project, four in Edmonton and three in Toronto. These focus groups included PPs, study personnel, clinic support personnel, organizational administrators, intervention and control physicians, other clinic physicians not involved in the project, and clinicians who worked in the clinic.

**Table 1:** Selected characteristics of study participants (N = 45)

**Characteristic**	**No. (%)**
Gender	
Male	5 (11.1)
Female	40 (88.9)
Profession	
Primary care physician	19 (42.2)
Managers	7 (15.7)
Registered nurse	5 (11.1)
Nurse practitioner	3 (6.7)
Quality coordinator	3 (6.7)
Administrative/clerical staff	2 (4.4)
Project staff	2 (4.4)
Pharmacist	1 (2.2)
Dietician	1 (2.2)
LPN	1 (2.2)
Social worker	1 (2.2)
Province	
Alberta	21 (47)
Ontario	24 (53)
Type of clinic	
Academic	26
Community	17
None	2
Main focus of role in project	
Research	4
Clinical	29
Administrative	9
Decision maker	2
Other	1

**Figure 1:**
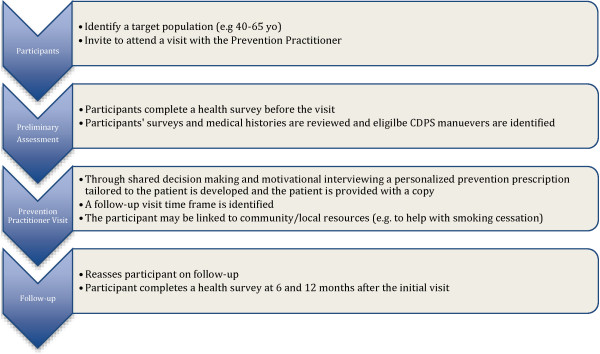
The BETTER chronic disease prevention and screening prevention practitioner intervention.

Participants highlighted that CDPS is not seen as a priority, nor is it integrated or remunerated in the present health care system. The BETTER Trial introduced a CDPS intervention into the practices (see Figure 1). The BETTER Trial was viewed as providing a framework for CDPS in a primary care system that often focuses on acute and chronic disease management rather than prevention. The results in this paper will focus on the theoretical framework that emerged as a possible solution to addressing CDPS in primary care.

*“The project brings a framework and an opportunity to spend time because we wouldn’t otherwise engage a patient in that process, it’s not remunerated you know”.* (Physician)

### The BETTER framework

**Figure 2:**
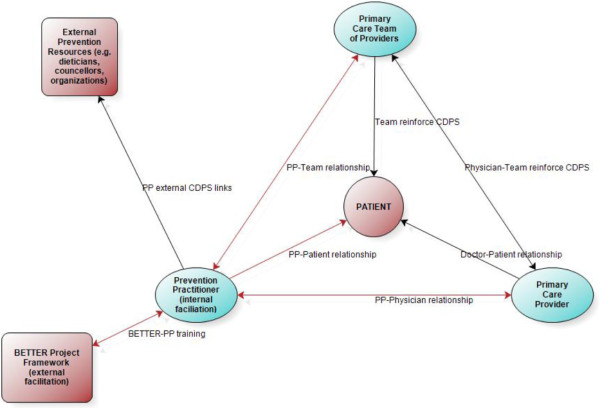
The BETTER chronic disease prevention and screening framework.

**Figure 3:**
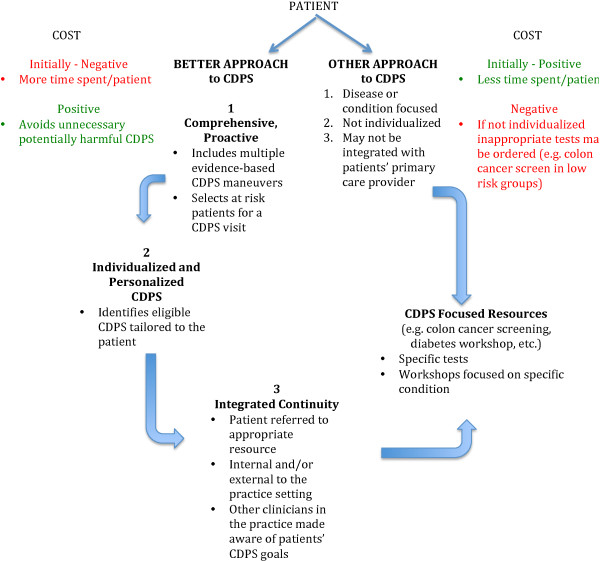
Key components of the BETTER approach.

Categories, themes and memos that emerged from the data were sorted and organized into a framework. The BETTER framework included (refer to Figure 2):

1) A newly developed role of PP with expertise in CDPS.

2) A unique combination of external and internal practice facilitation.

3) Key components, described below, that contribute to the success of CDPS (refer to Figure 3).

#### A novel role – the prevention practitioner (PP)

Participants viewed prevention and screening as an activity shared by many health care providers in the primary care setting. However it may not be well coordinated and no one has a comprehensive skill set in this area. Family physicians do not have time to address this in their day-to-day practice.

*“I guess the interesting thing is like to call yourself a primary Prevention Practitioner it’s not a protected term under like the regulated health professional block right and so it’s a, and like primary prevention falls within the scope of multiple professionals”.* (Pharmacist)

The PP was specifically trained to fulfill this new role and introduce the defined BETTER CDPS intervention into the practices (see Figure 1). Participants viewed this added skill set positively.

*“… the BETTER project because it you know had the educational component, and it had a lot of, it really helped us define your role, and a lot of you know what you do in a patient encounter, and what you do even when I’ve identified oh this patient has hyperlipidemia, can you please see them to have the discussion, you kind of inadvertently have this nice framework”.* (Physician)

#### External and internal practice facilitation

Practice facilitation has been described as a supportive service provided to primary care practices by a trained individual or team of individuals [35] and practice facilitators are specially trained individuals who work with primary care practices to make meaningful changes to improve patients’ outcomes [35]. A facilitator can be internal or external to a practice [35]. The BETTER Trial increased CDPS in the practice and the patients through a combination of external and internal practice facilitation.

### External facilitation

Originally we conceptualized the PP intervention of the BETTER Trial as only an internal practice facilitation intervention. However, it became apparent during the interviews and focus groups that the BETTER research trial itself provided external facilitation support to the intervention. The external facilitation included facilitating the introduction of the intervention at the sites and the training and supporting of the PP role.

Physician leads were identified at each site to facilitate the introduction of the PP to that site.

*“Dr. ____ is on our board and talked about the project with great enthusiasm and asked if we would support it …”* (Administrator)

Training and ongoing project support helped the PPs define and expand their new roles within the practice, and to develop the required skills to fulfill their new role. Regular teleconferences provided opportunities to share ideas and support one another in managing specific clinical situations.

*“a big part of the training was we did a lot of teleconference calls amongst the BETTER project team and all of the PPs so as we were starting into the project, as questions came up, it gave us all a chance to discuss how we were treating certain situations and you know exactly, the steps we were supposed to take so, that certainly evolved as we went along too”.* (PP)

External facilitation was more important at the beginning of the intervention to help develop the skills for PPs to fulfill their new role, and to assist the practices in adapting to the new PP role. Modifications were made as needed to ensure a good fit within each practice.

### Internal facilitation

The PP facilitated change within the practice through the introduction of skills, tools and resources for CDPS.

*“So it enhanced what we were doing. I mean helped even some of the guidelines she came up with, it was part of her tool kit”. *(Physician)

The PP transformed the practice to incorporate CDPS into daily practice activities. This enabled the practice to better support the patient in their individual CDPS goals. Change was accomplished through the PPs’ unique positions in the practices and their ability to develop relationships with the patients, the clinical teams and the physicians. The PPs developed relationships with patients that were distinct and unique from the doctor-patient relationship and, in some cases, patients disclosed information to the PP that they would not disclose in a group setting or even to their own physician.

*“The only thing with the group things though is people may not share their own personal blocks like reasons they are not doing it in a group the same way they would share things with the PP one on one and sometimes the PP might be listening to things that the patients are uncomfortable telling us about”.* (Physician)

*“The PP uncovered a lot more alcohol use”.* (Physician)

The PPs developed relationships with other members of their primary care teams that engaged the practice in CDPS at both a practice and a patient-level.

*“And so that enhanced ongoing care and, and the patients related to her as part of the team and then she was able to do the follow-ups and she could key me in on which patients had been through BETTER or she’d leave a note on the EMR to say you know this is some of things to discuss at the next appointment so that prompted me to address certain things that maybe the patient was already aware of and enhance that uptake for the patient”.* (Physician)

The PPs generated internal facilitation through the relationships with their team. This was crucial to sustaining the intervention and, once established, the PP role continued to incorporate CDPS in some of the practices, in some form, even after the BETTER Trial was completed.

*“But even so it, we saw them in the office after the BETTER Project and there’s that relationship built there and they do, we do, I do still bring it up if I recognize them from the BETTER Project I do pull up those goals that are scanned into their chart, we still do go over it because it’s just an ongoing support for them right”. *(PP)

The role of the PP combined with external and internal practice facilitation provided a framework for CPDS that transformed the practice through the use of relationships with someone internal to the practice as illustrated in Figure 2.

#### Key components

Key components that contributed to the success of the BETTER Trial were identified including (see Figure 3):

1. A comprehensive, pro-active, and holistic approach to CDPS that broadened the PP’s scope of practice.

*“I think as holistic front end screen is really important. I think it’s also a real big way to support inter-professional team practice because you can, you have the opportunity to look holistically at a patient in terms of what their needs might be and in terms of the kinds of services that you might offer”.* (Organization manager)

*“Well I just think as a model it’s you know, for a complimentary, collaborative team practice around stepping up roles, allowing people to work to scope or expanding scope and that was …an example of expanding scope in someone whose area of work is nutrition”.* (Physician)

2. An individualized and personalized approach at multiple levels tailored to the practice and the patient. The study fostered important relationships between the PP, patient, and practice as a whole, including physicians and other team members. For example, personal invitations sent to the patient from their physician helped to start a dialogue about CDPS and the support they could receive from their primary care team.

*“I mean we all had the protocol, we all had, you know the policies we had to do, but everybody did it their own way”.* (PP)

*“ It’s patient engagement and then you’re building a relationship and then it’s, I firmly believe that you get your best outcomes if, if you can achieve a positive outcome in patient care around prevention if you have trust and it has to be a personal relationship”.* (Physician)

3. Integrated continuity involved as a process of longitudinal continuity of information and personal relationships that integrated CDPS into the practice. The PP identified CDPS resources (e.g. dieticians for dietary advice), and when necessary, referred patients to resources within and outside the practice setting and where necessary referred patients to those resources. Other clinicians in the practice became better acquainted with those resources and began integrating them into their clinical activities. This improved utilization of internal and external CDPS resources. The PP followed-up with the patients’ goals and communicated plans with the entire team. When the patient saw other clinicians in the practice those clinicians were also aware of the patients’ CDPS goals and could provide further support to them to achieve their goals.

*“… we had this great opportunity as part of BETTER to have one of our team members you know get this training and expertise you need training and I guess the issues of what do we do about it now and how you know because it is a shared competency like how do we create a system where we aren’t doing piecemeal patient care but all team members feel optimized and, and as if their skills are being used as well”.* (Pharmacist)

4. Adaptable through a collaborative approach at the PP, practice, and patient-level.

*“I think it creates a stronger collaboration, it, it’s a better team connection in that the role becomes well defined, and very important. I suppose one that can be customized to different practices, people work in different ways and she’s, happens to be very flexible so, yeah it led to some strong collaboration that way”.* (Physician)

## Discussion

It has been estimated that it would take an additional 7.4 hours a day for a physician to address the US preventive services task force recommendations [36]. Our qualitative analysis also highlights a perceived lack of health care system support for CDPS in the family practice setting. It is clear that we need to approach CDPS in a different way. The BETTER Trial addressed this CDPS gap through a novel approach, building capacity in an existing member of the primary care team to have a new role as Prevention Practitioner with expertise in CDPS.

The BETTER Trial, informed by Ontario’s chronic disease framework [17], developed an integrated approach to CDPS that aimed to address different facets of the framework including health care organizations, practice teams and individuals and families. A clinical working group selected and harmonized the high-quality recommendations for the chronic conditions in the BETTER Trial [21]. The BETTER pragmatic cluster randomized controlled trial demonstrated that a unique intervention through a PP could improve the implementation of clinically important CDPS in a cost-effective manner [19]. This effective intervention directly focused on the patient through the use of a PP who met with the patient and through a process of shared decision making and motivational interviewing developed a prescription tailored to the patient.

Primary care requires expertise and roles that are different from the traditional biomedical model. For example, new roles such as patient navigators [37-39] have been developed to address gaps in chronic disease management. These new roles may have an impact on outcomes and effectiveness [39-41], and could reduce health disparities [41-44]. It is important to address CDPS in socially disadvantaged populations since the prevalence of chronic disease is higher than the general population [2,45]. The PP role may be able to address the CDPS gap through reduction of risk factors, and through a proactive approach, target the needs of vulnerable populations. There is evidence that modest changes in risk factor levels can bring about large improvements in health and thus reduce this burden of care [1].

The PP intervention had several key components. One was comprehensiveness, that is, the focus was on evidence based CDPS actions in patients 40–65 years of age, and not on a specific disease or condition. Focusing on the common modifiable risk factors of multiple chronic conditions is clearly more cost effective and comprehensiveness is important in enhancing equity in health [46]. Dr. Starfield’s work also demonstrates that health equity can be improved through person-focused prevention and improved coordination [46]. Both of these were key components of the BETTER Trial. We describe a new approach to integrated continuity that resulted in improved integration of CDPS into the practice. This was made possible through the continuity of the PP’s relationships with the patient, the physician and other members of the practice, and making links between internal and external CPDS resources and activities. This was how coordination was improved and how other clinicians in the practice became engaged in facilitating patients’ CDPS goals.

Further work is needed to determine if the BETTER approach can be adapted to other settings and should include patient perspectives. The main limitation of this project was that the practices involved in the project were located in urban centers and used EMRs. They could be seen as early adopters compared to those not using an EMR, and may tend to serve patients with a higher socioeconomic status as compared to practices in other locations such as inner city or rural. Hence the findings may not be transferable to those settings.

## Conclusions

The BETTER Trial Prevention Practitioner (PP) intervention impacts CDPS through a framework that involves external and internal practice facilitation using a novel role, the PP. The key components include: 1) approaching CDPS in a comprehensive and proactive manner, 2) an individualized and personalized approach at multiple levels, 3) integrated continuity of CPDS for the patient and within the practice, and 4) adaptability. The introduction of a novel role within the primary care practice with skills and expertise in CDPS may address gaps in prevention and screening.

## Abbreviations

CDPS: Chronic disease prevention and screening; PP: Prevention practitioner; EMR: Electronic medical record.

## Competing interests

The authors declare that they have no competing interests.

## Authors’ contributions

We certify that all individuals listed as authors of this manuscript: 1) have made substantial contributions to conception and design, or acquisition of data, or analysis and interpretation of data; 2) have been involved in drafting the manuscript or revising it critically for important intellectual content; 3) have given final approval of the version to be published; and 4) agree to be accountable for all aspects of the work in ensuring that questions related to the accuracy or integrity of any part of the work are appropriately investigated and resolved. DPM conceived and designed the study, conducted interviews in Alberta, analyzed and interpreted the data, and wrote the manuscript. MG contributed to the conception and design of the study, conducted interviews in Ontario, assisted with analysis and interpretation of the data, and assisted with drafting and writing the manuscript. JC contributed to the conception and design of the study, conducted interviews in Ontario, assisted with analysis and interpretation of the data, and assisted with drafting and writing the manuscript. GS contributed to the conception and design of the study, assisted with analysis and interpretation of the data, and assisted with drafting and writing the manuscript. AC contributed to the conception and design of the study, conducted interviews in Alberta, assisted with analysis and interpretation of the data, and assisted with drafting and writing the manuscript. JR contributed to the conception and design of the study, assisted with analysis and interpretation of the data, and assisted with drafting and writing the manuscript. JP contributed to the conception and design of the study, assisted with analysis and interpretation of the data, and assisted with drafting and writing the manuscript. CA contributed to the conception and design of the study, conducted interviews in Alberta, assisted with analysis and interpretation of the data, and assisted with drafting and writing the manuscript. RB contributed to the conception and design of the study, assisted with analysis and interpretation of the data, and assisted with drafting and writing the manuscript. SB contributed to the conception and design of the study, assisted with analysis and interpretation of the data, and assisted with drafting and writing the manuscript. EG contributed to the conception and design of the study, assisted with analysis and interpretation of the data, and assisted with drafting and writing the manuscript.

## Authors’ information

DPM, MG, JC, AC, EG are active family physicians and researchers with expertise in qualitative and quantitative methodologies. CA was a research coordinator in the BETTER Trial. JR has extensive experience with the evaluation and implementation of clinical practice guidelines. GS & JP are academic family physicians. RB & SB were Prevention Practitioners in the BETTER Trial.

## Pre-publication history

The pre-publication history for this paper can be accessed here:

http://www.biomedcentral.com/1471-2296/15/66/prepub

## Supplementary Material

Additional file 1 Interview guide. Click here for file
