# Supplementary material for: Finding a BETTER way: A qualitative study exploring the prevention practitioner intervention to improve chronic disease prevention and screening in family practice
Source: BMC Fam Pract. 2014 Apr 11;15:66. doi: 10.1186/1471-2296-15-66 (PMC4101848; doi:10.1186/1471-2296-15-66)
Supplement: Additional file 1 — Interview guide. [file 1471-2296-15-66-S1.docx]

Appendix 1

**Interview Guide**

BETTER: Qualitative Evaluation of the Intervention

I would like to thank you for participating in our interview for the BETTER Project. We appreciate that you have taken the timeout of your busy schedule to help us in our research efforts.

You may recall that the BETTER project involved two interventions. One was at the practice-level and the other was at the patient-level. For this interview we are interested in your impressions and experience with the patient-level intervention. The patient intervention consisted of a Prevention Practitioner from each practice setting who met with individual patients to develop a prevention prescription.

1. We are interested in your perceptions of the patient-level intervention in the BETTER project. Please reflect on your experience and tell us your story of the patient-level intervention from the beginning (for prevention practitioners that could include their experience with the training, etc)
   1. Identify and describe what worked well.
   2. Identify what could be done differently. That is, to identify and describe any barriers or obstacles including strategies that may address the hindrances.
   3. Identify the perceived benefits/overall value of the intervention
   4. Identify the perceived disadvantages of the intervention
   5. How did it impact your working relationships?
   6. How did the patient-level intervention impact the physicians, staff and patients of the clinic?
   7. Did the project impact patient flow? And how?
   8. Did patient have any comments about this intervention?
   9. How well were you prepared for the intervention – what worked & what could be improved on?
2. Based on your experience with the BETTER patient-level intervention, do you think it was beneficial or not?
   1. What would you suggest should be done differently?
   2. What would you recommend for the future
   3. If you felt it was beneficial, how would you ensure feasibility and sustainability for BETTER?
      1. How could a practice be organized to support a prevention practitioner?
      2. Did the clinic setting/perspective impact feasibility? If so, please describe how.
      3. Were there any financial impacts of this intervention?
      4. How did the prevention practitioner impact use of clinic space or time?
      5. How could this approach be financially feasible?
      6. How could this become a sustainable approach?
      7. What is the single most important impediment to implementation?
      8. What is the single most important impediment to continuation of this?
